# A defined anthocyanin mixture sourced from bilberry and black currant inhibits Measles virus and various herpesviruses

**DOI:** 10.1186/s12906-022-03661-7

**Published:** 2022-07-08

**Authors:** Rinu Sivarajan, Heike Oberwinkler, Valeria Roll, Eva-Maria König, Maria Steinke, Jochen Bodem

**Affiliations:** 1 Institute for Virology and Immunobiology, Julius-Maximilians-University of Würzburg, Versbacher Strasse 7, 97078 Würzburg, Germany; 2 Chair of Tissue Engineering and Regenerative Medicine, University Hospital Würzburg, Würzburg, Germany; 3 Fraunhofer Institute for Silicate Research ISC, Röntgenring 11, 97070 Würzburg, Germany

**Keywords:** Anthocyanin, Astaxanthin, Bilberry, Black currant, Herpesvirus, Measles virus

## Abstract

**Background:**

Anthocyanin-containing plant extracts and carotenoids, such as astaxanthin, have been well-known for their antiviral and anti-inflammatory activity, respectively. We hypothesised that a mixture of *Ribes nigrum L.* (Grossulariaceae) (common name black currant (BC)) and *Vaccinium myrtillus L.* (Ericaceae) (common name bilberry (BL)) extracts (BC/BL) with standardised anthocyanin content as well as single plant extracts interfered with the replication of Measles virus and Herpesviruses in vitro.

**Methods:**

We treated cell cultures with BC/BL or defined single plant extracts, purified anthocyanins and astaxanthin in different concentrations and subsequently infected the cultures with the Measles virus (wild-type or vaccine strain Edmonston), Herpesvirus 1 or 8, or murine Cytomegalovirus. Then, we analysed the number of infected cells and viral infectivity and compared the data to non-treated controls.

**Results:**

The BC/BL extract inhibited wild-type Measles virus replication, syncytia formation and cell-to-cell spread. This suppression was dependent on the wild-type virus-receptor-interaction since the Measles vaccine strain was unaffected by BC/BL treatment. Furthermore, the evidence was provided that the delphinidin-3-rutinoside chloride, a component of BC/BL, and purified astaxanthin, were effective anti-Measles virus compounds. Human Herpesvirus 1 and murine Cytomegalovirus replication was inhibited by BC/BL, single bilberry or black currant extracts, and the BC/BL component delphinidin-3-glucoside chloride. Additionally, we observed that BC/BL seemed to act synergistically with aciclovir. Moreover, BC/BL, the single bilberry and black currant extracts, and the BC/BL components delphinidin-3-glucoside chloride, cyanidin-3-glucoside, delphinidin-3-rutinoside chloride, and petunidin-3-galactoside inhibited human Herpesvirus 8 replication.

**Conclusions:**

Our data indicate that Measles viruses and Herpesviruses are differentially susceptible to a specific BC/BL mixture, single plant extracts, purified anthocyanins and astaxanthin. These compounds might be used in the prevention of viral diseases and in addition to direct-acting antivirals, such as aciclovir.

**Supplementary Information:**

The online version contains supplementary material available at 10.1186/s12906-022-03661-7.

## Background

During the last decades, direct-acting antiviral therapies against several human pathogenic viruses were developed. These therapies are usually virus-specific and show low activity against other viruses, while plant extracts can contain components, such as flavonoids active against a variety of different virus families.

Measles viruses belong to the negative-stranded RNA viruses. They are highly contagious human pathogens that cause a temporary immunodeficiency in the infected patients leading in cases to severe complications such as blindness, encephalitis, severe diarrhoea and related dehydration, ear infections, or acute respiratory infections such as pneumonia (for review see [1]). Measles virus infection still accounts for 4% of all deaths in children under age five worldwide. In some cases, the Measles virus can cause the fatal subacute sclerosing panencephalitis (SSPE) even decades after infection [2]. Although a potent vaccine is available, more than 500 Measles virus infections per year have been diagnosed from 2017 to 2019 in Germany, probably due to growing vaccination fatigue [3]. Thus, an antiviral agent that targets Measles virus replication and, thus, could have the potential to prevent the SSPE and other complications associated with the infection would be desirable.

Herpes viruses, such as Herpes simplex virus type 1 (HSV-1), Cytomegalovirus (CMV) and Human Herpesvirus 8 (HHV-8), are wide-spread pathogens with up to 80% seroconverters for HSV-1, up to 90% for CMV [4] and up to 87% for HHV-8 in Africa [5]. Herpesviruses belong to DNA viruses with a very stable genome. While all these viruses are generally well-controlled by the human immune system, Herpesviruses represent a thread to newborns and immunocompromised patients, such as organ recipients, where drug resistance-associated mutations are often observed [6]. Furthermore, congenital and perinatal CMV infection may lead to severe neurological symptoms resulting in microcephaly, jaundice, petechiae, hepatosplenomegaly, periventricular calcifications, choriinfluoretinitis, pneumonitis, hepatitis, and sensorineural hearing loss. Direct antiviral drugs, such as aciclovir, ganciclovir (GCV) and letermovir, can be used to reduce the severity of the clinical symptoms [7]. Still, GCV, foscarnet and cidofovir based therapies are often associated with adverse side effects. Especially the renal impairment of foscarnet may result in loss of the transplant in infected organ recipients.

The development of direct-acting antiviral drugs targeting viral entry, proteases and viral polymerases led to successful treatment regimes for human pathogens, such as Human Immunodeficiency virus 1 (HIV-1) [8], Herpesviruses [6], Hepatitis B and D [9] and C [10] viruses. However, the frequent occurrence of resistance-associated mutations, severe side-effects, and high therapy costs underline the need to discover new antiviral agents. Several plants, fruits and berry extracts have been described to show antimicrobial and antiviral properties or have been applied as nutraceuticals to prevent diseases in the past. For example, consumption of the carotenoid astaxanthin has been associated with cardiovascular disease or cataract prevention [11]. Furthermore, it inhibits *Helicobacter pylori*-induced inflammatory and oncogenic responses [12].

Various studies have reported that flavonoids inhibit the replication of viruses, such as Dengue virus (DENV) [13], Duck Hepatitis [14], Enterovirus 71 [15] and Influenza virus. Furthermore, anthocyanin-rich berry extracts have been reported to suppress HSV-1 in epithelial cells [16]. This anti-HSV-1-effect of flavonoid-rich plant extracts has been described for *Solanum melongena* L. peel extracts [17]. Anthocyanins derived from plants were shown to interfere with rotavirus adsorption to host cells [18]. Isolated anthocyanins from strawberry (*Fragaria vesca* L.) and raspberry (*Rubus idaeus* L.) of the *Rosaceae* plant family, and *Vaccinium myrtillus* L. (Ericaceae) (Common name bilberry) (BL), and lingonberry (*Vaccinium vitis-idaea* L.) were shown to suppress Coxsackievirus B1 and Influenza replication [19]. Moreover, it has been reported that delphinidin, an anthocyanin contained, for example, in *Ribes nigrum*, L. (Grossulariaceae) (common name black currant) (BC), can inhibit the attachment stage of enveloped ( +) ssRNA viruses like Hepatitis C virus, West Nile virus, DENV, Zika virus and DNA viruses such as HSV-1. Cyanidin-3-sambubiocide was reported to bind to the viral neuraminidase of the Influenza virus and block virus entry [20].

In the present study, we aimed to analyse whether a mixture of BC and BL extracts (BC/BL) prepared under GMP (food) conditions (Figs. S1 to S5) with standardised anthocyanin content (30%) and astaxanthin interfered with the replication of the Measles virus (RNA-virus) and various Herpesviruses (DNA viruses) and thus could be used in the prevention or in treatment of various viral diseases. We investigated whether these extracts inhibited viral replication and cell-to-cell spread by qPCR and infectivity assays. Furthermore, we sought to characterise the BC/BL components responsible for the antiviral effects leading to the description of a novel, well-characterized antiviral substances.

## Methods

### Cell culture

All cell culture plates were obtained from Greiner Bio-One GmbH (Frickenhausen, Germany). Cells were cultured in Dulbecco's Modified Eagle Medium (DMEM, Thermo Fisher Scientific, Schwerte, Germany) complemented with 10% fetal calf serum and Penicillin/Streptomycin (Sigma-Aldrich, Taufkirchen, Germany). Vero SLAM cells were provided by S. Schneider-Schaulies (University of Würzburg). All other cell lines (Vero, BHK-21, MDCK, NIH 3T3 and HepG2) were purchased from ATCC (LGC Standards GmbH, Wesel, Germany).

### Viruses

The recombinant green fluorescent protein (gfp*)*-encoding Measles, murine Cytomegalovirus (mCMV), HHV-8 and HSV-1 viruses were obtained from J. Schneider-Schaulies (*gfp*-encoding *wild-type* and vaccine Measles strains) (Virology, Würzburg), Lars Dölken (HSV & mCMV) (Virology Würzburg, Germany), Wolfram Brune (HHV-8) (Source: Brune, HPI Hamburg, Germany), used by permission.

### Plant extracts and compounds

The mixture of BC/BL extracts (from *Ribes nigrum* L. and *Vaccinium myrtillus* L., respectively) were obtained from Medpalett AS (Anthocyanin mix, source Medpalett AS, Healthberry 865®, batches S-080415, S-170418) and the single BL (batch R8214801) and BC extracts (batch J23119005) from Medpalett AS (Sandnes, Norway). The identification of the anthocyanins in the extracts was performed with qualitative high-performance liquid chromatography (HPLC) analysis and UV absorbance using internal standards as described before (Figs. S1**-**S3) [21]. The extracts contain a minimum 28–30% of total anthocyanins. The experiments were repeated with extracts from berries harvest in a different year. Our analyses revealed that the 5 anthocyanins described in the paper (C3R/C3G/D3R/D3G/PT3G) were present at concentrations of 0.5–5% (Figs. S1-S4). Cyanidin-3-glucoside (Kuromanin chloride, C3G, purity: > 97%), cyanidin-3-rutinoside (Keracyanin chloride, C3R, purity: > 97%), delphinidin-3-glucoside chloride (D3G, purity: > 97%), delphinidin-3-rutinoside chloride (D3R, purity: > 97%) and petunidin-3-glucoside (PT3G, purity: > 97%), were provided by Polyphenols AS, Biolink Group (Sandnes, Norway). Astaxanthin (Asta) (purity: ≥ 97%) and aciclovir were obtained from Sigma-Aldrich. All compounds were dissolved and diluted in the cell culture medium (DMEM), except for Asta, which was solubilised in dimethylsulfoxide (DMSO Sigma-Aldrich). The respective solvents served as controls in cytotoxicity and viral inhibition assays. All the plant names have been checked with http://www.theplantlist.org.

### Cellular toxicity

To exclude any toxic side effects of the used compounds, cell survival and metabolism were measured by RealTime-Glo™ MT Cell Viability Assay (Cat. No. G9712, Promega, Mannheim, Germany). Vero SLAM, BHK21, MDCK, NIH 3T3, and HepG2 cells (2 × 10^4^) were incubated with decreasing amounts of the compounds solubilised in DMEM or DMSO. Wells with either DMEM alone or DMSO served as controls. The RealTime-Glo™ MT Cell Viability Assay was performed according to the manufacturer's instructions in triplicates. After 1 h, and then every six or 12 h, the luminescence was measured with a Centro LB 960 microplate luminometer (Berthold Technologies). Changes in the luminescence over time were calculated and compared to the solvent control. Only non-cytotoxic concentrations of every compound were used for antiviral assays.

### Cellular proliferation assays

The BC/BL extract and the single BC and BL extracts used in the present study have a dark blue to violet colour, which might influence the luminescence readouts of cellular cytotoxicity assays such as the RealTime-Glo™ MT Cell Viability Assay. Thus, for these extracts, the proliferation of cells was also determined by direct automatic cell counting. Cells were seeded on optical plates (CellCarrier-96, PerkinElmer) and counted before the experiments. Then the compounds were added in decreasing concentrations, and the cells were incubated for three days. The medium was changed, and cell numbers per well were determined using the PerkinElmer Ensight reader. Only compound concentrations that did not reduce the cell number per well significantly were used for antiviral assays.

### Measles virus replication assay

Vero SLAM cells were incubated with decreasing concentrations of the solubilised compounds for approximately 1 h. The assays were performed in black 96 well plates (PerkinElmer GmbH, Rodgau, Germany) at least in three independent experiments. Cells were infected with *gfp*-encoding wild-type Measles virus (MOI: 0.5) and incubated for two days. Due to the autofluorescence, the medium was replaced by DMEM, and *gfp*-expressing cells were subsequently quantified using a TECAN saphire2 plate reader. Every well was measured five times at 25 spots (six-well plates) or nine places (96-well plates) utilising the beam settings "fine" and "sensitive". Autofluorescence of a plate with untreated and non-infected cells was measured, and the mean background fluorescence intensity was subtracted from the values obtained above.

Direct counting of infected cells: Vero SLAM (*wild-type*) or Vero cells (Edmonston vaccine strain (MOI: 0.5)) were seeded in optical 96-well plates and infected with *gfp*-encoding Measles viruses. One day after infection, Measles virus-infected cells and *gfp*-expressing cells were directly counted using the PerkinElmer Ensight system with optical cell culture plates in triplicates. The instrument was controlled by manual counting. Viral titers were calculated as infectious particles per ml. Three independent experiments were performed. Changes in viral infectivity were analysed by the Student's t-test, comparing the values at a single concentration with the solvent control.

### HSV-1, mCMV and HHV-8 infections

BHK-21 cells (HSV-1 and HHV-8) or NIH 3T3 cells (mCMV) were incubated with decreasing concentrations of the solubilised compounds for approx. 1 h. All concentrations were analysed by six (HSV-1) or three (HHV-8, mCMV) independent replicates on black 96 well plates. Cells were infected with *gfp*-encoding *wild-type* HSV-1 (MOI: 2.5), *gfp*-encoding wild-type HHV-8 (MOI: 0.5) or *gfp*-encoding mCMV (MOI: 0.5). One day (HSV-1) or two days after infection (HHV-8 and mCMV), infected cells and *gfp*-expressing cells were directly counted using the PerkinElmer Ensight system with optical cell culture plates. The instrument readout was controlled by manual counting. For infection studies with HSV-1, aciclovir served as the positive control. Changes in viral infectivity were analysed by the Student's t-test, comparing the values at a single concentration with the solvence control.

## Results

### The BC/BL extract inhibits Measles virus infection, cell-to-cell spread, and syncytia formation

**Fig. 1:**
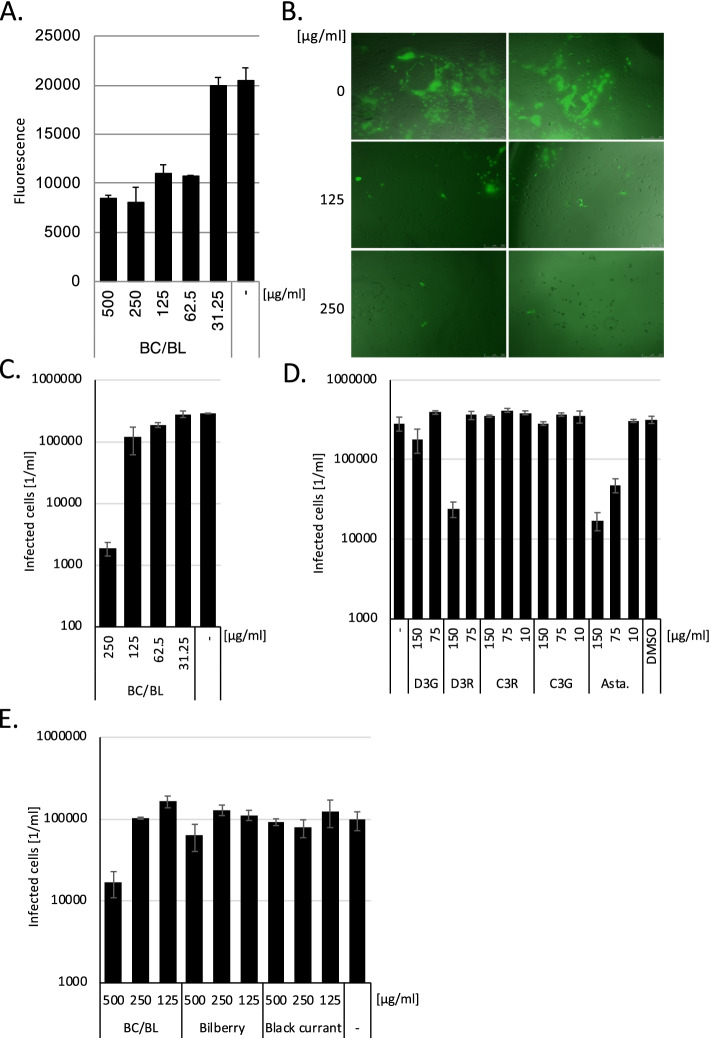
Impact of BC/BL extract, anthocyanins and astaxanthin on Measles virus infection. **A**. Determination of the inhibition of *gfp*-encoding Measles virus infection by BC/BL. Each bar represents the mean of 3 independent infection experiments. Error bars represent the standard deviation. **B**. Immunofluorescent analysis of BC/BL on *gfp*-encoding Measles virus inhibition and syncytia formation. **C**. Quantitative analysis of *gfp*-positive Vero SLAM cells after BC/BL treatment and Measles virus infection. Data are presented as means (bars) and standard deviation (error bars) of three independent samples performed in triplicates, each. **D**. Impact of astaxanthin (Asta) and D3R on *gfp*-encoding Measles virus replication. Data are presented as means (bars) and standard deviation (error bars) of four independent samples performed in quadruplets, each.** E**. Impact of BC/BL and single extracts on *gfp*-encoding Measles virus infection. Data are presented as means (bars) and standard deviation (error bars) of three independent samples performed in triplicates, each. The significance of the results was calculated, and the values are shown in the main text

Since the development of antiviral drugs against the Measles virus is still unsuccessful, we sought to analyse whether the defined BC/BL extract (Healthberry 865®) inhibited Measles virus replication. These extracts are normalized on the content of antocyanins. We treated Vero SLAM cells with decreasing BC/BL extract concentrations and subsequently infected the cells with a *gfp*-encoding wild-type Measles virus (Fig. 1A). The BC/BL was still soluble at 500 µg/ml concentration, while concentration of 1 mg/ml showed precipitates after centrifugation. In the selected concentration range, the extract did not show any effect either on cell growth or cellular metabolism (data not shown). The virus-containing medium was removed after 1.5 h. Then the medium was replaced by a medium containing the compound at the same concentration to remove the virus from the supernatant, and the cells were further incubated for one day. Since our preliminary results indicated that the compound exhibits a strong auto-fluorescence at the GFP excitation/emission wavelength, the fluorescence was measured through the bottom of the plate using a fluorescence plate reader (Fig. 1A). First, non-infected and non-treated cells were measured to compensate for the auto-fluorescence of the cells. The statistical mean of these measurements was subtracted from the fluorescence values. We found that 500, 250, 125, and 62.5 µg/ml of the BC/BL extract efficiently inhibited Measles virus replication to 41.5, 39.5, 53.7 and 52.4%, respectively, compared to the non-treated control group, whereas 31 µg/ml of the BC/BL extract had no effect. This indicates that BC/BL contains antiviral components active against *wild-type* Measles virus.

We sought to further study BC/BL's effect on the Measles virus replication by analysing the infection by fluorescence microscopy. Vero SLAM cells were seeded on optical plates, treated with the BC/BL extract at 250 and 125 µg/ml, and infected with *gfp*-expressing wild-type Measles virus. Compared to untreated cells, BC/BL efficiently inhibited viral replication at 250 µg/ml (Fig. 1B). Furthermore, it blocked the cell-to-cell spread and syncytia formation nearly completely. The lower concentration of 125 µg/ml still showed some inhibition of infection and prevented the formation of large syncytia typical for Measles virus infection (Fig. 1B). The observed inhibition of cell-to-cell infection might be the key to efficiently blocking virus spread in infected patients since the Measles virus is known to be strongly cell-associated. Moreover, the reduction of infected cells indicates that the BC/BL extract targets replication steps before genome replication, such as receptor recognition/binding, entry, uncoating or viral transcription. Moreover, comparing the inhibition data with the microscopic images, it seems reasonable to assume that the extract's autofluorescence leads to higher fluorescence readings at higher concentrations, which would lower the measured inhibition by BC/BL. Since the fluorescence measurement in this experiment depends on the number of infected cells on the one hand and the expression levels on the other, we decided to count the infected cells directly.

Vero SLAM cells were seeded on optical plates to measure the infected cells by their gfp-expression directly. BC/BL was added, and the cells were infected with *gfp*-encoding *wild-type* Measles virus in triplicates. The next day, the *gfp*-expressing cells were counted (Fig. 1C) using the PerkinElmer Ensight system, which allows to precisely determine the number of *gfp*-expressing infected cells per well. Again, BC/BL reduced the number of infected cells significantly (250 µg/ml 153fold (significance *p* = 3*10^–8^)), even at concentrations as low as 62.5 µg/ml (significance 1.5fold, *p* = 0.001) (Fig. 1C), providing evidence that the BC/BL extract acts prior to gene expression. Since BC/BL is a mixture of black currant and bilberry extracts, we analysed whether both extracts contribute to the inhibition. Vero SLAM cells were incubated with BC/BL, or extracts from BC and BL alone and subsequently infected with *gfp*-encoding *wild-type* Measles virus (Fig. 1E). After one day, infected *gfp*-expressing cells were counted as described above. Neither BL nor BC extracts alone reduced viral infection, indicating that the preparation of BC/BL might have prevented the degradation of the active components such as anthocyanins. All experiments were repeated with BL/BC extracts from harvested in a different year with comparable results. In summary, the BC/BL extract contains an antiviral component against the *wild-type* Measles virus, which can block syncytia formation.

### Delphinidin-3-rutinoside chloride and astaxanthin inhibit Measles viral replication

BC/BL contains various anthocyanins, which could contribute to the inhibition of Measles viruses. Next, we wanted to determine the active components of BC/BL. Thus, infection experiments with the purified anthocyanins D3G, D3R, C3R, C3G, and Asta, a carotenoid, were performed (Fig. 1D). Only non-toxic compound concentrations were applied. The analyses were performed with decreasing concentrations, starting with 150 µg/ml since these concentrations are comparable to the 30% anthocyanin content of BC/BL. The compounds were added directly before the cells were infected since anthocyanins are unstable in water-based solutions at physiological pH. DMSO was added as solvent control since Asta is poorly soluble in water and was thus dissolved in DMSO. The infection experiments were performed in quadruplets. Asta and D3R reduced the number of infected cells at concentrations of 75 µg/ml (Asta, significance *p* = 0.003) and 150 µg/ml (Asta (significance *p* = 0.0002) and D3R (significance *p* = 2.9*10^–5^)), by one order of magnitude. At the same time, the other anthocyanins did not influence Measles viral replication. These data indicate that Asta and D3R contribute to the inhibition of Measles virus infection by BC/BL.

### BC/BL does not inhibit the Edmonston vaccine strain

To further analyse the mechanism and the specificity of the inhibition of Measles virus by the extract BC/BL, Measles virus vaccine strain Edmonston was used. Measles viruses require SLAM (CD150) as the cellular entry receptor. The viral hemagglutinin protein (H) binds with its head domain to a beta-sheet of the membrane distal ectodomain of the CD150 receptor [22]. In contrast, the Measles vaccination strain Edmonston can use CD46 as a cellular receptor [23]. This fundamental difference provides an opportunity to analyse whether the inhibition of the Measles virus by BC/BL is due to a specific receptor-dependent viral entry block. Thus, the entry of the vaccination strain should not be affected if BC/BL targets viral entry in a SLAM dependent way.

**Fig. 2:**
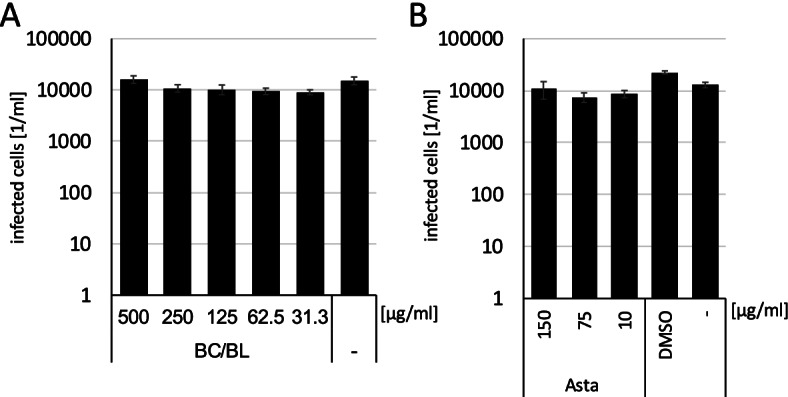
Impact of BC/BL (**A**) and astaxanthin (**B**) on the Edmonston vaccine strain. Bars represent the mean of infected cells from six independent wells. Error bars show the standard deviation. Each experiment was performed in quadruplets. Virus infection with the Edmonston strain was not reduced significantly by the treatment (calculated with the Student’s t-test)

Wild-type Vero cells, without CD150/SLAM receptor, were infected with a recombinant *gfp-*expressing Edmonston strain in the presence of either BC/BL (Fig. 2A) or with astaxanthin (Fig. 2B) since we have shown that astaxanthin blocks *wild-type* Measles virus entry as well (Fig. 1D). Both BC/BL and astaxanthin did not influence viral infection significantly. This finding indicates that components of BC/BL and Asta interact either with the wild-type H-protein or block the SLAM receptor specifically. Furthermore, neither BC/BL, Asta, nor D3R inhibit any of the Measles viruses' downstream steps since these pathways are similar in the wild-type and vaccine virus. In summary, we have shown that the BC/BL extract and astaxanthin can only impede *wild-type* Measles virus, while the vaccine strain is unaffected.

### BC/BL inhibits HSV-1 infection

**Fig. 3:**
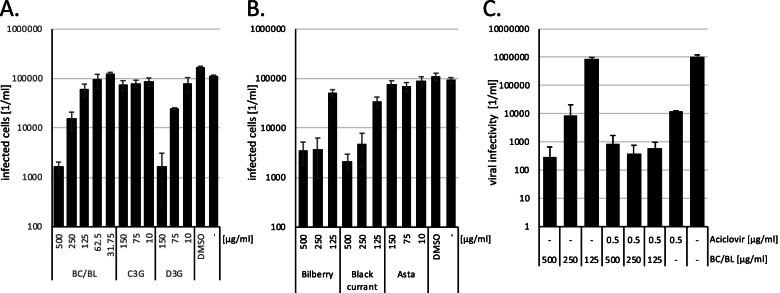
Impact of BC/BL, single anthocyanins and astaxanthin on HSV-1 infection. **A**., **B**. Quantitative analysis of infected BHK-21 cells after treatment with extracts or compounds and infection with *gfp*-encoding HSV-1. **C**. Analysis of viral infectivity after cell treatment with BC/BL and aciclovir. All the data (**A**-**C**) are presented as means (bars) and standard deviation (error bars) from six independent wells. The significance of the analyses was calculated, and the values are shown in the main text

Having shown that BC/BL inhibits a negative-stranded RNA virus, we sought to investigate whether BC/BL would inhibit other unrelated human pathogentic DNA viruses. Since chronic viral infections caused by herpes viruses are common and antivirals without severe adverse side effects are still needed, we decided to analyse whether BC/BL or astaxanthin inhibited HSV-1 and further identify the active antiviral component of the BC/BL extract. Only non-toxic compound concentrations were applied. BHK-21 cells were pre-incubated with either BC/BL or single extracts from bilberry or black currant, starting with decreasing concentrations from 0.5 mg/ml. Thus, similar concentrations of the potential active compounds were used. The cells were subsequently infected with *gfp*-encoding HSV-1 at a multiplicity of infection of 2.5, and infected *gfp*-expressing cells were counted one day after infection using the PerkinElmer Ensight system. BC/BL (500 µg/ml) suppressed viral infectivity about two orders of magnitude (*p* = 0.001) (Fig. 3A). The concentrations of 250 and 125 µg/ml reduced the number of infected cells by 7.3 (significance *p* = 0.001) and 1.8 (significance *p* = 0.001) fold, respectively. In comparison, extracts from black currant and bilberry alone inhibited viral infection more than factor 42 and 26, respectively (significance black currant *p* = 1.0*10^–8^; significance bilberry *p* = 1.3*10^–8^), indicating that HSV-1 infection is inhibited significantly (Fig. 3B).

Next, we sought to identify the active component in BC/BL by analysing the effect of D3G, D3R, C3R, and C3G on HSV-1 infection. Additionally, we included Asta. BHK-21 cells were pre-incubated with the anthocyanins or Asta at decreasing concentration, starting from 150 µg/ml and infected with *gfp*-encoding HSV-1. The number of infected cells was determined as described above. While C3G (Fig. 3A), Asta (Fig. 3B), D3R, and C3R (data not shown), did not influence HSV-1 infection, D3G inhibited infection by two orders of magnitude (significance *p* = 6.7*10^–8^) at a concentration of 150 µg/ml, indicating that D3G is an active compound in the extract (Fig. 3A). This result provides evidence that the suppression of HSV-1 is virus-specific since Asta was inactive against HSV-1. Furthermore, we show that an extract containing multiple anthocyanins, such as BC/BL, can efficiently inhibit several different viruses.

HSV-1 infections are usually treated with aciclovir. Thus, we compared the susceptibility of our HSV-1 isolate to BC/BL with the inhibition by aciclovir. BHK-21 cells were inhibited with BC/BL, aciclovir, and a combination of BC/BL and aciclovir and infected with *gfp*-expressing HSV-1. Since aciclovir inhibits DNA replication, we analysed the infectivity of the generated viruses after a single round of infection. Cell culture supernatants were collected after two days and used to infect fresh BHK-21 cells. After one additional day, *gfp*-expressing cells were counted. Again, BC/BL inhibited the infection. However, by analysing viral infectivity after a complete-single round, we observed a suppression of more than three orders of magnitude, showing that BC/BL efficiently suppresses the replication of HSV-1 (Fig. 3C). Furthermore, this indicates that BC/BL treatment might affect additional replication steps downstream of the early gene expression. Moreover, we show that the inhibition of HSV-1 by aciclovir is independent of BC/BL since both 125 and 250 µg/ml of BC/BL plus aciclovir suppressed viral infectivity more effectively than the compounds alone. In addition, this indicates that the combination of BC/BL with aciclovir might be beneficial for infected patients and might allow the use of lower aciclovir concentrations.

### BC/BL inhibits mCMV infection

**Fig. 4:**
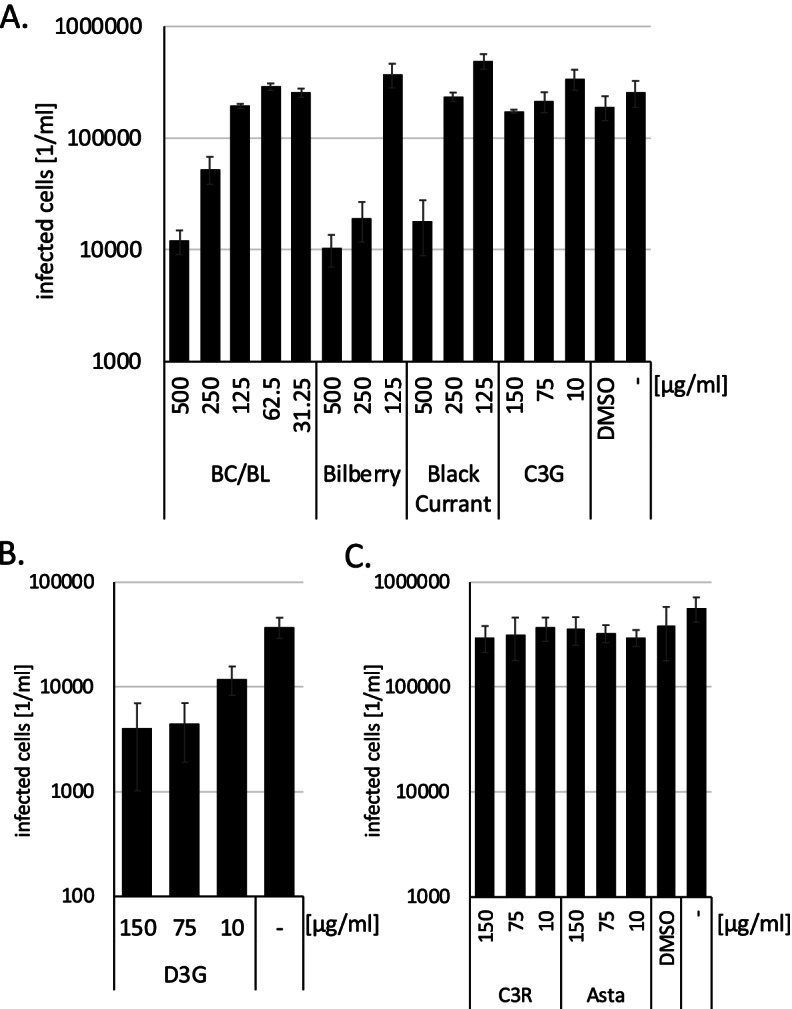
Impact of BC/BL, single anthocyanins and astaxanthin on mCMV infection. **A-C**. Quantitative analysis of infected NIH 3T3 cells after treatment with extracts or compounds and infection with *gfp*-encoding mCMV. Data are presented as means (bars) and standard deviation (error bars) from three independent experiments. The significance of the results was calculated, and the values are shown in the main text

Having observed the suppression of HSV-1 infection by BC/BL, we sought to analyse whether BC/BL would inhibit the related Cytomegalovirus (herpesvirus 5). The *gfp*-expressing mCMV was chosen as a model virus. NIH 3T3 cells were pre-incubated with decreasing concentrations of either BC/BL, bilberry, or black currant extracts, starting with a concentration of 0.5 mg/ml. Thus, similar concentrations of the potential active compounds in the extracts were used. Only non-cytotoxic concentrations were used. The cells were infected with *gfp*-encoding mCMV, and infected *gfp*-expressing cells were quantified two days after infection with the PerkinElmer Ensight reader (Fig. 4). Similar to our HSV-1 results, BC/BL inhibited mCMV infectivity emphasising that BC/BL acts as a general inhibitor of herpes viruses. Both BL and BC extract separately suppressed mCMV by twofold (significance *p* = 0.0002) and onefold (significance *p* = 0.0031), similar to BC/BL (twofold, significance *p* = 0.0002) at a concentration of 500 µg/ml (Fig. 4A). However, the inhibition of viral infection with mCMV was lower than the suppression of HSV-1.

To identify the mCMV suppressing compound of BC/BL, C3R, C3G, and D3G effects on mCMV infections were analysed. Our experimental results with the Measles virus prompted us to analyse whether Asta was effective in these analyses as well since Asta might interfere with the receptor. NIH 3T3 cells were incubated with D3G, C3G, Asta, or C3R starting from 150 µg/ml and subsequently infected with *gfp*-expressing mCMV (Fig. 4). After two days, infected cells were quantified using the Ensight system. Again, neither C3R, C3G, nor Asta influenced viral infectivity. At the same time, D3G reduced viral infection by about one order of magnitude at a concentration of 150 and 75 µg/ml (significance *p* < 0.004), indicating that D3G might be a component generally active against Herpesviruses.

### BC/BL inhibits human herpesvirus 8 infection better than single bilberry or black currant extracts

**Fig. 5:**
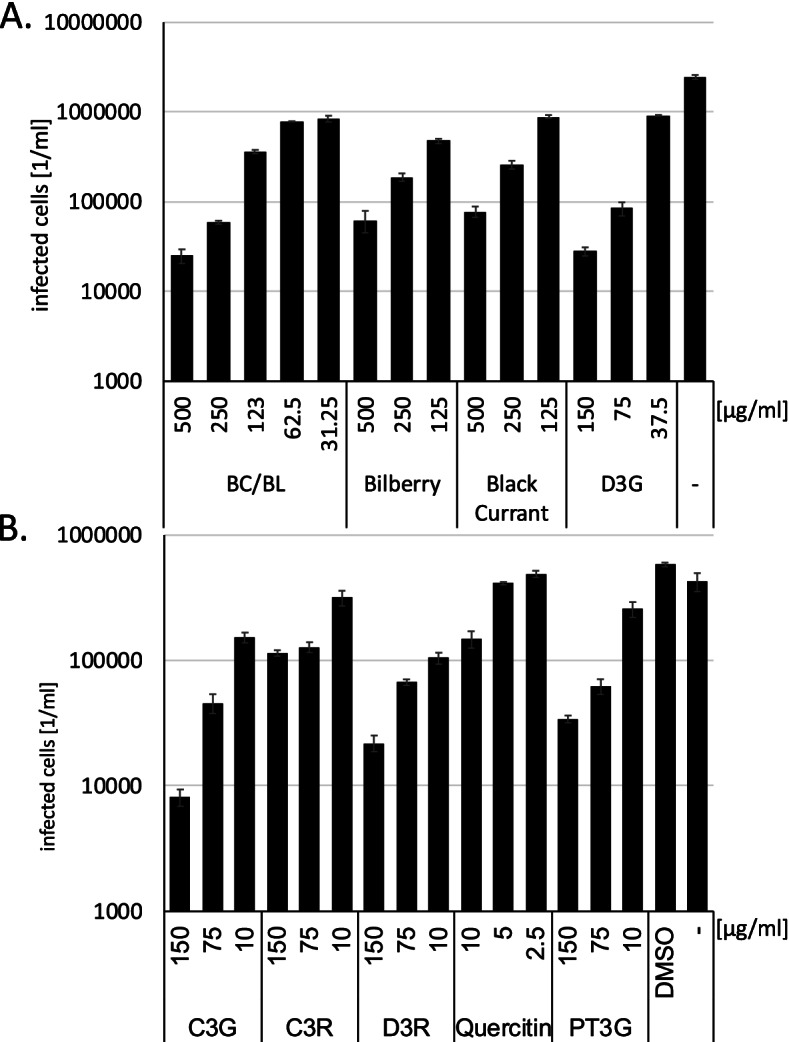
Impact of BC/BL and single anthocyanins on HHV-8 infection. **A**., **B**. Quantitative analysis of infected BHK-21 cells after treatment with extracts or compounds and infection with *gfp*-encoding HHV-8. Data are presented as means (bars) and standard deviation (error bars) from three independent experiments. The significance of the results was calculated, and the values are shown in the main text

Since we have shown that BC/BL suppresses HSV-1 and mCMV we decided to analyse its influence on the Human herpesvirus 8 (HHV-8), which can lead to fatal Kaposi-Sarcoma. Thus, we analysed whether BC/BL would inhibit virus replication. BHK-21 cells were pre-incubated with decreasing concentrations of either BC/BL, bilberry, or black currant extracts starting with 0.5 mg/ml. Only non-cytotoxic concentrations were used. The cells were infected with *gfp*-encoding HHV-8, and infected *gfp*-expressing cells were quantified two days after infection with the PerkinElmer Ensight reader (Fig. 5A). Similar to our results with HSV-1, BC/BL inhibited viral infectivity by two orders of magnitude at the highest concentrations indicating the BC/BL acts as a general inhibitor to Herpesviruses. Furthermore, both bilberry and black currant extracts inhibited HHV-8 less than BC/BL, indicating a synergistic effect of both extracts in BC/BL. The observed reductions with the extracts were highly significant (significance *p* < 1*10^–6^).

To further identify potential HHV-8 inhibiting components, BHK-21 cells were incubated with different purified anthocyanins and subsequently infected with HHV-8. Infected cells were counted. D3G and C3G suppressed viral infection by approximately two orders of magnitude (significance *p* < 3*10^–8^) (Fig. 5). C3R showed only minor effects (reduction approximately threefold at 150 and 75 µg/ml, significance *p* < 4*10^–6^), while PT3G and D3R inhibited viral infection in more than one order of magnitude. All observed viral infectivity reductions, except C3R at 10 µg/ml, were significant with *p* < 0.002. In summary, BC/BL, the single BL and BC extracts, D3G, and C3G were effective against HHV-8. Our results provide evidence that BC/BL inhibits both RNA and DNA viruses specifically.

## Discussion

Although vaccines represent the gold-standard in viral disease prevention, growing vaccination fatigue and unavailability of vaccines result in frequent outbreaks of infectious diseases, even in developed countries. Plant-derived therapeutics could fill the demand for cost-effective, available drugs with fewer adverse side effects. Here, we have shown that a specific combination of BL and BC extracts, standardised to an anthocyanin content of 30%, can have a role as a plant-derived antiviral, and so may serve in the prevention of severe viral diseases, such as Measles and Herpesvirus infections. Furthermore, the suppression of Measles virus infection by two orders of magnitude is within the range of other antiviral drugs, so that these extracts might be used to prevent the diseases allowing to limit outbreaks. However, identifying the BC/BL's active components was desirable, first to define the concentration and second to use the purified anthocyanins as therapeutic in higher doses.

While the antiviral characteristics of anthocyanins have been already described, the influence on Measles virus infection has never been reported before. Furthermore, the specific inhibition of the wild-type virus entry by BC/BL is remarkable (Figs. 1 and 2). The Measles virus's sensitivity to D3R indicates that this compound, a major constituent anthocyanin present in BC/BL, might contribute to the berry extract's antiviral effect. Since the achieved viral inhibition by D3R is within the range of the suppression by BC/BL, D3R is likely the extract's main anti-Measles virus factor. Anthocyanins from Elderberry (*Sambucus nigra* L.) have been used to inhibit influenza viruses [24]. However, the observed suppression of viral replication was less than 40%, which indicates that either influenza viruses are not sensitive to anthocyanins or the concentration was too low.

Here, we have shown that BC/BL and Asta influence the infection with the Measles wild-type virus and not with the Edmonston vaccine strain (Figs. 1 and 2). Asta is known to act against oxidative stress and spans cellular membranes. The latter points out that it may interact with the SLAM receptor blocking virus entry. Asta could interrupt SLAM multimerisation, which could be studied in a future project. However, the flavonoid compound baicalin was shown to block HIV-1 entry, obviously using a similar approach as BC/BL in blocking the receptor interaction [25]. Previously, it has been shown that Asta blocks the uptake of the human papillomavirus (HPV) L1 protein into the sperm membrane [26]. Unfortunately, a direct antiviral effect against HPV has not been reported so far.

To our knowledge, this is the first study describing a receptor-dependent effect of both a specific combination of berry extracts (BC and BL) and astaxanthin on virus uptake. This effect's specificity is remarkable since it would still allow the replication of the Edmonston vaccine strain. Thus, treatment or prevention of Measles infections with BC/BL or astaxanthin would not interfere with Measles vaccination.

Surprisingly, BC/BL has the broad ability to inhibit HSV-1, mCMV and HHV-8. Since BC/BL is an extract of berries without known adverse side effects, it might be beneficial for seropositive patients in the prevention of Kaposi-Sarcoma in immunocompromised HHV-8 and CMV associated diseases. The achieved suppression of viral replication with more than 3 orders of magnitude in a single-round experiment is in the range of direct-acting viral drugs used in HIV-1 treatment (Fig. 3). Comparing our results with the inhibition of HSV-1 by chebulagic acid (CHLA) and punicalagin (PUG), two hydrolyzable tannins were isolated from Terminalia chebula Retz. [27], anthocyanins benefit from their low toxicity and suppress HSV-1 more than these polyphenolic secondary metabolites. The analyses of the anthocyanins inhibiting different herpesviruses revealed that D3G was a general inhibitor of this virus group. This indicates that D3G should be further evaluated for its ability to suppress herpesviruses in vivo and that it might serve as lead compound for the development of new drugs (Figs. 3 to 5). Furthermore, since the D3G mediated suppression of HSV-1 and HHV-8 infection was comparable to BC/BL, indicating that D3G might be the active BC/BL component. D3G suppressed mCMV weaker, indicating that BC/BL also contains other active components. Our analyses of the anthocyanins active against HHV-8 revealed that C3G, PT3G, and D3R block HHV-8 efficiently and indicate that stabilised D3G forms could be used in treatment.

## Conclusions

Asta and defined anthocyanins from a combination of BC and BL extracts are active against an array of unrelated DNA and RNA viruses in a virus specific manner. These extracts have been used as food supplements without known adverse side-effects and could, thus, be safely used in the prevention of viral diseases. The effective concentrations with antiviral properties in the present study are in the low mg/ml range (≤ 0.5 mg/ml). Furthermore, we showed that D3G acts a inhibitor for Herpes virus replication and D3R suppresses Mealses virus indicating that both substances can be used as lead-compound for further developments [28].

## Supplementary Information

**Additional file1: Figure S1. **HPLC profile of BC/BL extracts (Lot. no. S-170418). **Figure S2. **HPLC analysis and profile of BC/BL extracts (Lot. no.S-080415). **Figure S3.** HPLC analysis and profiles of the black currant extract. **Figure S4. **HPLC analysis and profiles of the bilberry extract. **Figure S5.** GMP-statement for BC/BL

## Data Availability

The datasets used and/or analysed during the current study are available from the corresponding author on reasonable request.

## Abbreviations

Asta: Astaxanthin
BC: Black currant
BC/BL: Mixture of black currant / bilberry extracts
BL: Bilberry
C3G: Cyanidin-3-glucoside
C3R: Cyanidin-3-rutinoside
CMV: Cytomegalovirus
D3G: Delphinidin-3-glucoside chloride
D3R: Delphinidin-3-rutinoside chloride
DENV: Dengue virus
DMEM: Dulbecco's Modified Eagle Medium
DMSO: Dimethylsulfoxide
GCV: Ganciclovir
gfp: Green fluorescent protein
H: Viral hemagglutinin
HHV-8: Human Herpesvirus 8
HIV-1: Human Immunodeficiency virus 1
HPLC: High performance liquid chromatography
HSV-1: Herpes simplex virus type 1
mCMV: Murine Cytomegalovirus
PT3G: Petunidin-3-glucoside
SSPE: Subacute sclerosing panencephalitis
