# Supplementary material for: A defined anthocyanin mixture sourced from bilberry and black currant inhibits Measles virus and various herpesviruses
Source: BMC Complement Med Ther. 2022 Jul 8;22:181. doi: 10.1186/s12906-022-03661-7 (PMC9264518; doi:10.1186/s12906-022-03661-7)
Supplement: Supplementary file 1 — Additional file1: Figure S1. HPLC profile of BC/BL extracts (Lot. no. S-170418). Figure S2. HPLC analysis and profile of BC/BL extracts (Lot. no.S-080415). Figure S3. HPLC analysis and profiles of the black currant extract. Figure S4. HPLC analysis and profiles of the bilberry extract. Figure S5. GMP-statement for BC/BL [file 12906_2022_3661_MOESM1_ESM.docx]

**HPLC/UV methods and material quality**

The identification of the anthocyanins in the extracts was performed with qualitative HPLC analysis (Slimestad and Solheim, 2002) and UV absorbance using internal standards as described before (Figures S1-S4) (Chen and Breen, 1991; Porter et al., 1985; Price and Bulter, 1977). We analysed the content of 5 anthocyanins described in the paper (C3R/C3G/D3R/D3G/Pet-3-G) and found concentrations of 0.5-5% (Figure S1 to S4).

**Figure S1: HPLC profile of BC/BL extracts (Lot. no. S-170418)**

**Figure S2: HPLC analysis and profile of BC/BL extracts (Lot. no. S-080415).**

**Figure S3: HPLC analysis and profiles of the black currant extract.**

**Figure S4: HPLC analysis and profiles of the bilberry extract.**

Furthermore, regarding quality of the extracts, we provide information from the GMP statement from the Norwegian food authority and add: The BC/BL extracts is compliant to the specification for E163, Anthocyanins, as listed in Reg. (EU) No. 231/2012, laying down specifications for food additives.


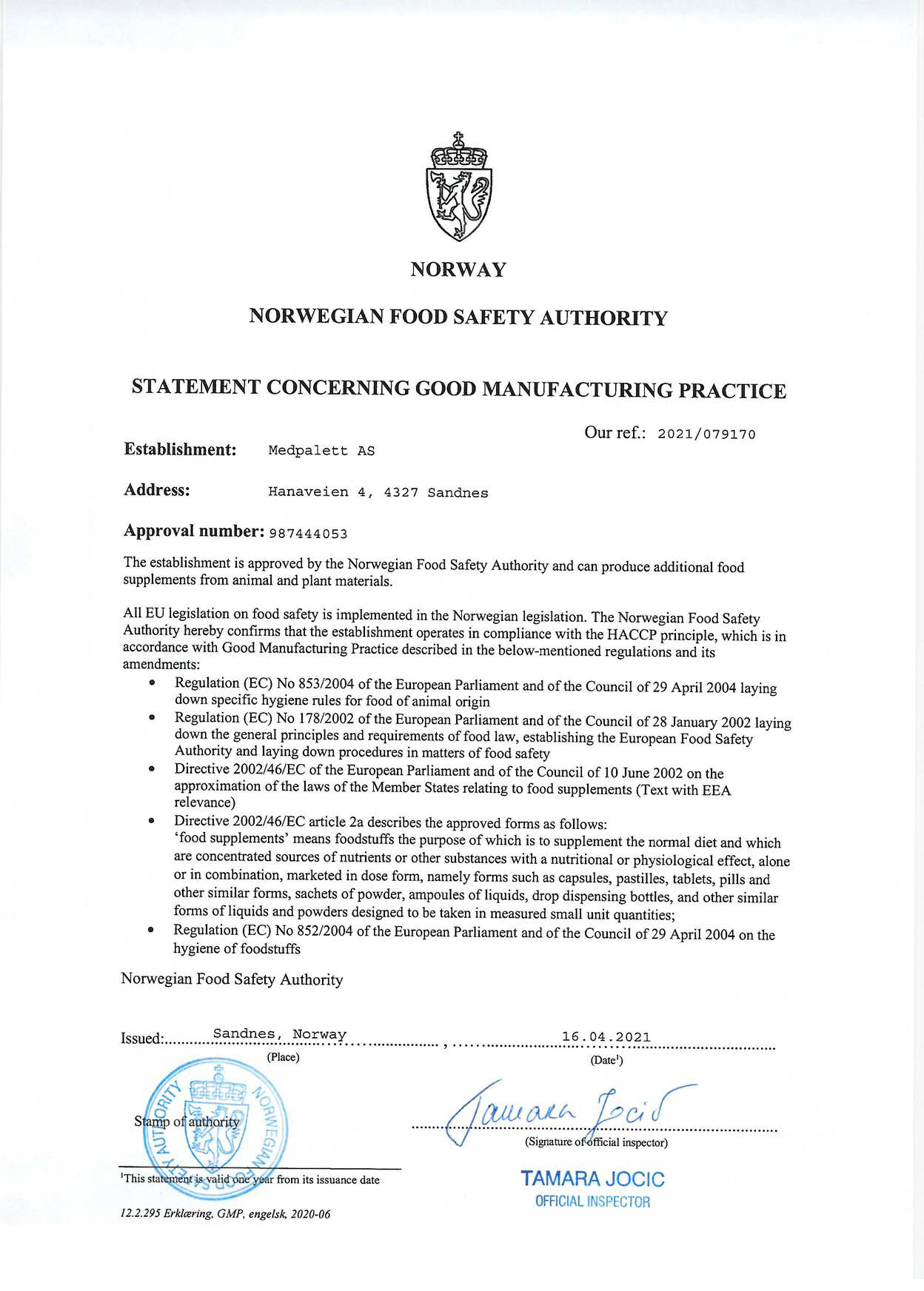


 Figure S5: GMP-statement for BC/BL

Chen, G.C., Breen, P.J., 1991. Activity of Phenylalanine Ammonia-Lyase (PAL) and Concentrations of Anthocyanins and Phenolics in Developing Strawberry Fruit. J. AMER. Soc. HORT. SCL 116, 865-869.

Porter, L., Hrstich, L.N., Chan, B.G., 1985. The conversion of procyanidins and prodelphinidins to cyanidin and delphinidin. Phytochemistry 25, 223-230.

Price, M.L., Bulter, L.G., 1977. Rapid visual estimation and spectrophotometric determination of tannin content of sorghum grain. Agricultural and Food Chemistry 25, 1268-1273.

Slimestad, R., Solheim, H., 2002. Anthocyanins from black currants (*Ribes nigrum* L.). J Agric Food Chem 50, 3228-3231.
